# Impact of free access to leisure facilities and community outreach on inequalities in physical activity: a quasi-experimental study

**DOI:** 10.1136/jech-2017-209882

**Published:** 2018-01-12

**Authors:** James Higgerson, Emma Halliday, Aurora Ortiz-Nunez, Richard Brown, Ben Barr

**Affiliations:** 1 Department of Public Health and Policy, University of Liverpool, Liverpool, UK; 2 Division of Health Research, Faculty of Health and Medicine, Lancaster University, Lancaster, UK; 3 Leisure and Environment Department, Blackburn with Darwen Borough Council, Blackburn, UK

**Keywords:** physical activity, policy, inequalities

## Abstract

**Background:**

There are large inequalities in levels of physical activity in the UK, and this is an important determinant of health inequalities. Little is known about the effectiveness of community-wide interventions to increase physical activity and whether effects differ by socioeconomic group.

**Methods:**

We conducted interrupted time series and difference-in-differences analyses using local administrative data and a large national survey to investigate the impact of an intervention providing universal free access to leisure facilities alongside outreach and marketing activities in a deprived local authority area in the northwest of England. Outcomes included attendances at swimming and gym sessions, self-reported participation in gym and swim activity and any physical activity.

**Results:**

The intervention was associated with a 64% increase in attendances at swimming and gym sessions (relative risk 1.64, 95% CI 1.43 to 1.89, P<0.001), an additional 3.9% of the population participating in at least 30 min of moderate-intensity gym or swim sessions during the previous four weeks (95% CI 3.6 to 4.1) and an additional 1.9% of the population participating in any sport or active recreation of at least moderate intensity for at least 30 min on at least 12 days out of the last four weeks (95% CI 1.7 to 2.1). The effect on gym and swim activity and overall levels of participation in physical activity was significantly greater for the more disadvantaged socioeconomic group.

**Conclusions:**

The study suggests that removing user charges from leisure facilities in combination with outreach and marketing activities can increase overall population levels of physical activity while reducing inequalities.

## Background

Physical inactivity is linked to a wide range of physical and mental health outcomes^1^ and is estimated to cost the National Health Service (NHS) £455 million a year.^1^ There is a steep socioeconomic gradient in physical activity in the UK with 76% of men in the highest income quintile achieving recommended physical activity levels compared with only 55% of men in the lowest quintile.^2^ Increasing levels of physical activity in more disadvantaged groups could improve overall population health and reduce health inequalities.

Reducing the cost of participation is one potential means to increase physical activity and address these inequalities. Local government and charitable organisations in England provide a range of leisure services, including swimming, gym and other sports facilities. The provision of these facilities is generally subsidised to promote social inclusion, community well-being^3^ and public health.^4^ Some local authorities (LAs) have sought to increase levels of physical activity and promote public health by removing user charges entirely, offering free access to leisure facilities.^5–7^ Evaluation of such schemes is important to ensure that they are reaching their target audience. For example, free leisure offers could inadvertently increase inequalities in participation if they are mainly used by those already active or more affluent. Analysis of uptake of the national free swimming initiative in Bristol found no relationship between area deprivation and participation.^8^ Public health goals are, however, often not the main aim of pricing policies, with income generation frequently a key competing concern.^9^

Some studies comparing participation rates before and after the introduction of free offers have reported increases in participation,^10–16^ while another found that increased charges were associated with a small decline in participation.^17^ There is some evidence that respondents in surveys do not report that entrance charges are a major barrier to participation, and that this is just one of many factors that influence their participation.^10^ These studies, however, provide limited evidence of the likely impact that community-based initiatives involving free access to leisure centres have on physical activity levels.

We therefore investigated the impact of the re:fresh scheme, introduced in Blackburn with Darwen, a deprived LA in the northwest of England in 2008, that provided free access to activities in leisure centres (swimming pools and gyms) at most times of the day along with community outreach activities. We use quasi-experimental methods to investigate whether the scheme led to an increase in swimming and gym activities and overall levels of physical activity and whether these effects differed by socioeconomic group.

## Methods

### Setting

Blackburn with Darwen is a deprived and ethnically diverse LA, in the northwest of England with a population of 147 489. In 2015, Blackburn with Darwen was ranked as the 24th most deprived area out of all 326 lower-tier LAs in England and 31% of the population were from a black or minority ethnic group.

### Intervention

The re:fresh scheme began in July 2008, with the provision of free access to local government leisure facilities at most times of the day for people living, working or registered with a general practitioner in Blackburn with Darwen. At the time, there were nine leisure facilities in Blackburn with Darwen. Three of these facilities included swimming pools and gyms, one facility just had a swimming pool and five sites had gym facilities only. A map showing the distribution of these facilities in relation to area deprivation is given in online supplementary appendix 1. Several of the leisure facilities were located close to deprived neighbourhoods. Initially in July 2008 the free offer was only available to people >50 years old, being extended to people aged 16–24 years in September 2008 and finally to people aged 25–49 years in April 2009. Overall during the scheme, free leisure was available for 90% of the opening hours of the nine facilities.

10.1136/jech-2017-209882.supp1 Supplementary file 1

The free offer was supported by outreach work delivered by Health Trainers and a Healthy Communities Partnership. Five full time equivalent (FTE) Health Trainers were employed during the project, offering 1 to 1 and group sessions, to around 700 inactive people per year supporting behaviour change through goal setting and motivational interviewing. Two FTE community workers delivered the Healthy Communities Partnership which supported a network of volunteers who ran community events to engage people in taster sessions and increase the awareness of re:fresh, and act as buddies to accompany people to their first activity sessions.

The programme was also supported by considerable marketing and promotional activity to raise awareness of the offer and to promote participation. The scheme is ongoing, although in 2016 a flat fee of £1 was introduced for previously free activities in response to cuts in local government funding. The scheme was jointly funded by the NHS and Blackburn with Darwen Borough Council. Between 2008 and 2014, the NHS contributed a total of £6 million on top of the core funding for leisure facilities provided by the council over this period (£22 million). The outreach activities cost approximately £2 million over this time.

### Datasets

Our analysis used two datasets. First, we extracted data from the leisure management IT system for Blackburn with Darwen providing data on every attendance at a leisure centre from 2005 to 2014. This dataset was then used to calculate the total quarterly number of gym and swim attendances from 2005 to 2014 and the proportion of these that were free (ie, there was no cost associated with them on the leisure management system).

Second, we used data from a large national annual survey of sports participation—the Active People Survey (APS). The methodology for this survey is described elsewhere.^18^ This cross-sectional telephone survey is based on a random sample from each LA in England selected using Random Digit Dialing. One person aged ≥16 is randomly selected from eligible household members. Average response rates are low, ranging from 27.1% to 27.8% during the study period. We therefore applied survey weights in all our analysis. Respondents are asked to report the number of days in the past four weeks they have engaged in sports and other active recreation, including gym and swimming sessions, for at least 30 min and the intensity of these activities. The interviews for each survey are evenly spread across 12 months, running from October of one year to October of the next year. We used data for all surveys from APS1 (2005–2006) to APS9 (2014–2015). There was a gap from October 2006 to October 2007 when no survey was completed. We pooled all data from these nine surveys giving a total pooled sample of 1 763 780 individuals aged ≥16. Data in the sample were missing on age for 2.2%, ethnicity for 1.8% and socioeconomic status for 2.2%. A further 7.4% of the sample was excluded as their socioeconomic status was unclassifiable based on their reported occupation. Excluding these data provided a sample of 1 556 563 for the analysis, 6160 of which was within Blackburn with Darwen and 1 550 403 from the other LA areas of England.

### Outcomes

Our analysis included three outcomes. First, the relative change in the number of gym and swim attendances (combined) at Blackburn with Darwen leisure centres before and after the introduction of the re:fresh scheme (outcome 1). Second, the proportion of people reporting in the APS that they had engaged in at least 30 min of moderately intensive gym or swimming activity in the past four weeks (outcome 2). Third, the proportion of people reporting in the APS they had engaged in any sport or active recreation of at least moderate intensity for at least 30 min on at least 12 days out of the last four weeks (outcome 3). Outcome 3 was designated as a national indicator in 2008 by the government for measuring the performance of LAs at promoting health and well-being and increasing participation in sport.

### Analysis

First, we used data extracted from the Leisure Management System, to conduct an interrupted time series (ITS) analysis investigating the relative change in attendances associated with the introduction of re:fresh. ITS is a quasi-experimental method using data from multiple time points before and after an intervention in order to detect whether or not the intervention had a significantly greater effect than any underlying secular trend.^19^ We use data on the number of attendances for the 14 quarters (ie, 3-month periods) before the intervention and 26 quarters after the intervention in a log-linear regression model with Newey-West estimators to account of autocorrelation in the data. We log transformed the data to aid interpretation of the coefficients as relative change in activity; that is, relative risk (RR). We additionally included time trend terms for before and after the intervention and dummy variables for the four quarters of the year to adjust for seasonal changes. We used Newey-West automatic bandwidth selection procedure^20^ to estimate the maximum lags required to take into account the autocorrelation structure of the data (further details are given in online supplementary appendix 2). The effect of the re:fresh programme was estimated by including a dummy variable indicating the period after the introduction of the re:fresh scheme in the third quarter of 2008 (see online supplementary appendix 2 for full model formula). To investigate whether there was a different effect on swimming compared with gym attendances, we additionally replicated the ITS analysis separately for gym and swimming attendances. In sensitivity analysis, we replicated models with untransformed count data and using a Poisson regression model rather than linear regression. (see online supplementary appendix 2).

Second, we used APS data to conduct a difference-in-difference^21^ analysis comparing the change in outcomes within Blackburn with Darwen to the change in the rest of England, before (2005–2007) and after (2008–2014) the re:fresh intervention. This difference-in-differences approach accounts for both national trends in our outcomes and unobserved time-invariant differences between Blackburn with Darwen and the rest of the country that could confound findings. The difference between the change in outcomes within Blackburn with Darwen and the change in outcomes in the rest of the country—known as the difference-in-difference parameter—provides an unbiased estimate of the intervention effect if the trends in outcomes would have been parallel in Blackburn with Darwen and in the rest of the country in the absence of the re:fresh programme.^21^ We used a linear regression model including a dummy variable indicating the intervention area (Blackburn with Darwen) and a dummy variable indicating the before (2005–2007) and after (2008–2014) periods. The interaction term between these two variables is the difference-in-differences parameter. Although our outcome is binary, we use linear regression as this interaction term cannot be interpreted as the programme effect in non-linear models, and linear probability models provide an unbiased estimate of the difference-in-differences parameter even with a binary outcome.^22^ We additionally included variables to control for changes in the composition of the population over time—age, age squared, sex, ethnicity (white British, white other, Asian, Black, Chinese, mixed, other) and three socioeconomic groups based on the National Statistics Socioeconomic Classification (managerial and professional, intermediate and routine/manual/never worked/long-term unemployed). We repeated the analysis removing the ‘never worked’ category as a sensitivity analysis (see online supplementary appendix 3). We included a time trend to account for the national secular trend and used survey weights to adjust for non-response. We estimated robust SEs clustered at the LA level to allow for within LA correlation due to sampling design. We repeated the difference-in-differences analysis for both outcome 2 (swim and gym activity) and outcome 3 (any physical activity). To investigate whether there was a differential effect across socioeconomic groups, we additionally carried out the analysis separately for each socioeconomic group. As a sensitivity test we conducted the difference-in-differences analyses using alternative comparison groups (the most deprived 20% of LAs, deprived LAs outside London, deprived LAs outside London with high black and ethnic minority populations and other deprived LAs in the northwest). We also replicated the analysis with the intervention start date set as 2009, rather than 2008 to address the inclusion of some preintervention data using 2008–2009 survey data (see online supplementary appendix 3). To investigate whether there was a different effect on swimming compared with gym attendances, we also repeated the analysis separately for gym and swimming participation.

## Results

### ITS analysis

**Figure 1:**
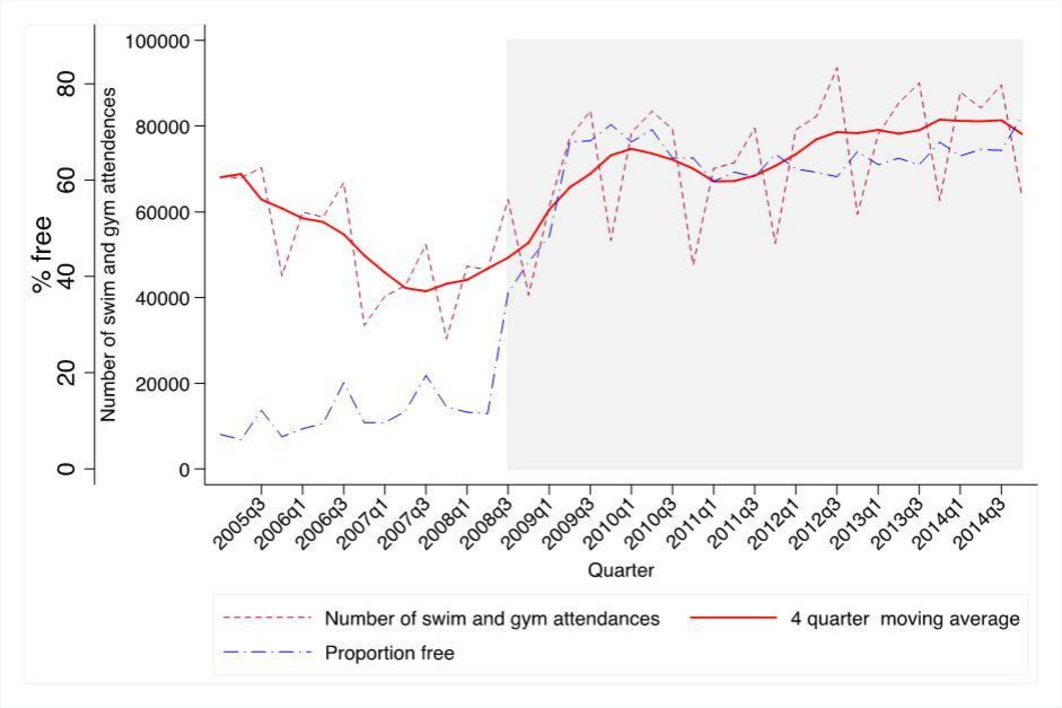
Quarterly trend in swimming and gym attendances in Blackburn with Darwen before and after the introduction of re:fresh and the proportion of all attendances that were free. Source: Leisure Management System.

Figure 1 shows that 11% of gym and swimming attendances were free before the intervention; this increased to 63% after the intervention. The trend in gym and swimming attendances was declining before the intervention, and this trend reversed in line with the introduction of the intervention.

The ITS regression indicated that the introduction of re:fresh was associated with a 64% increase in gym and swimming activity (RR 1.64, 95% CI 1.43 to 1.89, P<0.001). This equates to an additional 26 400 additional swim and gym attendances per quarter due to the re:fresh initiative over the 2008–2014 period. Additional analysis shown in online supplementary appendix 4 indicated a larger effect size when analysing the effect on gym attendances alone; models using alternative specifications showed similar results (see online supplementary appendix 4).

### Difference-in-differences analysis

**Figure 2:**
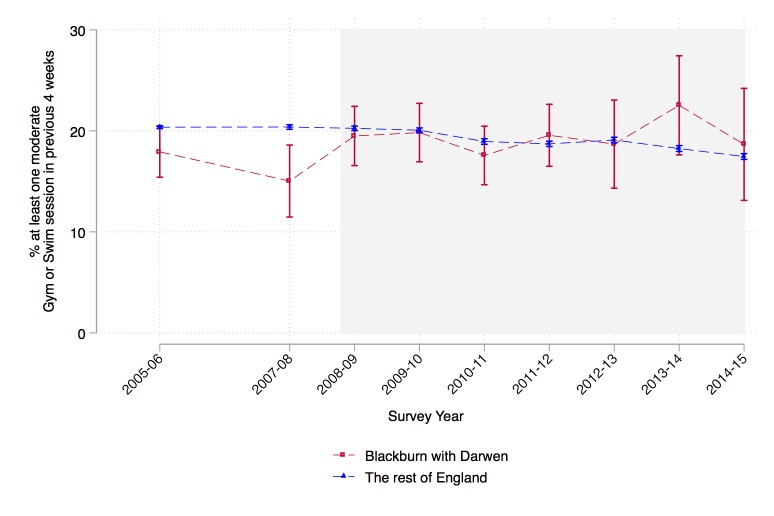
Trend in the proportion of people reporting at least one moderate gym or swim session in the previous four weeks, 2005–2014 in Blackburn with Darwen and the rest of England. Source: Active People Survey.

Figure 2 shows the trend in participation in gym and swimming activities reported in the APS for Blackburn with Darwen and England as a whole. While there was a slight drop in activity between 2005 and 2007 in Blackburn with Darwen, this was not significant. Following the introduction of re:fresh, there was an increase in gym and swimming participation, while the national rate was constant before the introduction of re:fresh in 2008 and fell slightly after this point.

**Figure 3:**
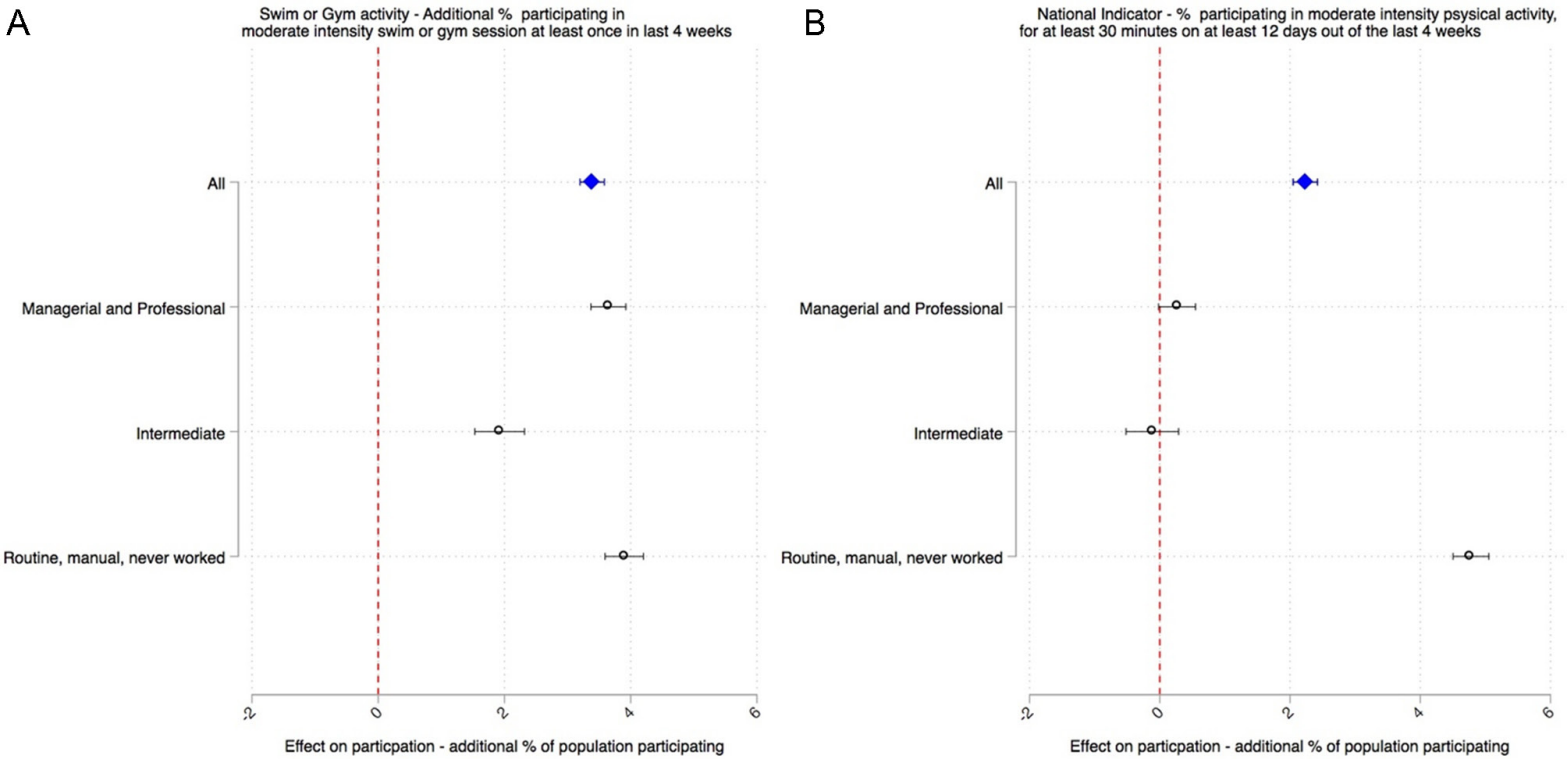
Estimates of the effect of the introduction of re:fresh from the difference-in-differences analysis on (A) % participating in gym or swim activity at least once in the past month and (B) the % participating in any sport or active recreation for a least 30 min on at least 12 days over the last four weeks. Results for all socioeconomic groups in Blackburn with Darwen and separately for three socioeconomic groups. Effect sizes indicate the additional percentage of the population participating due to the intervention.

Figure 3 shows the estimates from the difference-in-differences analysis. The intervention was associated with an additional 3.9% of the population in Blackburn with Darwen participating in at least 30 min of moderate-intensity gym or swim sessions during the last four weeks (95% CI 3.6 to 4.1). This effect was greater in the most disadvantaged socioeconomic group (4.7%, 95% CI 4.4 to 5.0), followed by managerial and professional groups, and the effect was lowest in the intermediate socioeconomic group. In terms of overall participation in physical activity, the intervention was associated with an additional 1.9% of the population participating in any sport or active recreation of at least moderate intensity for at least 30 min on at least 12 days out of the last four weeks (95% CI 1.7 to 2.1). This effect was much larger in the more disadvantaged routine and manual group (3.6%, 95% CI 3.3 to 3.8) and was not significant in the more advantaged socioeconomic groups. In online supplementary appendix 5, we provide participation rates by socioeconomic group before and after the intervention showing that within Blackburn with Darwen inequalities narrowed after the intervention, while they remained relatively unchanged nationally. In relative terms, compared with average levels of participation before the intervention this is equivalent to a 20% increase in the proportion of people participating in at least 30 min of moderate-intensity gym or swim sessions in a month (95% CI 19% to 21%) and an 8% increase in the proportion participating in any sport or active recreation for a least 30 min on at least 12 days over the last four weeks (95% CI 7% to 9%).

Sensitivity analyses given in online supplementary appendix 3 show similar results using alternative comparator groups when using 2009 as the intervention start date and when removing the ‘never worked’ category from the socioeconomic classification. Replicating the analysis separately for the proportion of people reporting gym and swim sessions in the past four weeks indicated that for swimming effect sizes were similar across socioeconomic groups while the effect on gym activity was significantly higher among people from routine and manual groups compared with professional, managerial and intermediate groups (see online supplementary appendix 3).

## Discussion

### Main finding of this study

We found that the introduction of a scheme providing widespread free access to leisure facilities alongside outreach activities led to an increase in swimming and gym attendances at these facilities, an increased proportion of the population participating in swimming or gym activity and increased overall physical activity levels. The increases in participation were greatest in the most disadvantaged socioeconomic group—decreasing inequalities.

In relative terms, the intervention had a lower effect on overall levels of physical activity (8% increase) than on swimming and gym activity (20% increase), suggesting that there may have been some substitution of activities; that is, that some people may have shifted from other activities to gym and swimming, without necessarily increasing levels of overall physical activity.

Larger effects were seen for gym attendances at LA facilities than swimming attendances although effects were similar for the additional number of people participating in gym activities compared with swimming. We also found very little effect of the intervention on the numbers of people participating in any gym activity from more affluent socioeconomic groups. One possible explanation is that there was some shifting from private gyms to LA gyms when the latter became free, particularly among more affluent groups. This would have increased the number of gym attendances in LA gyms while not increasing the numbers of people participating in any gym activities.

### What is already known on this topic

Very little is known about the effectiveness of community-wide public health programmes to improve physical activity rates.^15 23–26^ The only studies to our knowledge investigating the impact of community-based initiatives involving free access to leisure centres have been based on simple before and after comparisons, or retrospective surveys asking respondents to recall their participation prior to the intervention or asking respondents hypothetical questions about whether they would have participated in the absence of the intervention.^10–14 16 24 25^ While these studies have generally found that these interventions were associated with increased participation, all of these approaches are likely to be highly susceptible to bias and provide limited evidence for the impact of the interventions. There have been a number of systematic reviews of the impact of providing financial incentives to individuals to increase physical activity, including free membership of leisure facilities.^26–28^ These concluded that providing unconditional financial incentives has little effect on physical activity. Providing free access to leisure facilities across a population, however, may have a different effect from targeting free membership at particular inactive individuals.

### What this study adds

This is the first study to our knowledge that uses quasi-experimental methods to investigate the impact on physical activity of a community-wide scheme to offer free access to leisure facilities to the whole population alongside outreach activities. This study suggests that this approach is effective at increasing overall levels of physical activity and reducing inequalities in physical activity.

### Strengths and limitations

Our study has a number of strengths. First, by using a consistent dataset of attendances at leisure centres over multiple time periods spanning a decade we were able to use ITS analysis methods to estimate effects while accounting for any secular trends in the data. This provides a more robust analysis than a simple before and after comparison; however, it may still be subject to bias if there were other unobserved determinants of physical activity that changed around the same time as the intervention. Second, by using a difference-in-differences analysis we were able to account for any change in national trends around the same time as the intervention as well as any unobserved time-invariant differences between Blackburn with Darwen and the rest of the country that could confound findings. While we also controlled for observed changes in the composition of the population, some risk of confounding remains if there were other unobserved determinants of physical activity that only changed in Blackburn with Darwen around the same time as the intervention and not in other LAs. Third, our analysis is strengthened by finding consistent results across multiple outcomes and datasets. These include both objective measures and those based on self-reported estimates, and they ranged from outcomes more proximal to the intervention—attendance at a swimming pool or gym—to wider population measures of physical activity.

A number of limitations however remain. Measuring attendance at swimming or gym sessions using transaction data may be subject to error. People may not be captured on the system if they enter the facilities without swiping their membership card, or decide not to attend the activity initially logged on the system, or move between activities within a leisure facility. These errors could lead to bias if the level of error changed before and after the intervention. Self-report in surveys will be subject to biases in reporting and recall. Validation studies of self-reported questionnaires have shown inconsistent results compared with more robust methods.^29^ The APS it a telephone-based survey with a low response rate, which may affect the validity and reliability of the data. Although we adjusted for known correlates of non-response using survey weights, response bias could still be a problem, particularly if those most active are more likely to participate in the survey because they are engaged with the subject. To investigate this further, we compared reported levels of participation in the APS to those using similar questions in the Health Survey for England, a face-to-face survey with a higher response rate (60%). We found very similar levels of reported activity in both surveys, suggesting that the low response in the APS is not leading to bias in estimates of overall participation (see online supplementary appendix 6). Due to the nature of our analysis, response bias would only influence our overall findings if there was a change in the groups more likely to respond over time in Blackburn with Darwen, which was not reflected in the sample from other LAs. Our use of both objective transaction data and more subjective survey data aimed to address uncertainties associated with each data type, with both indicating significant increases in participation.

As outlined above, while the free offer was a substantial part of the intervention it also included outreach and marketing activities that were targeted at inactive groups. In our analysis, we are not able to distinguish between the effects of these different components and can only measure the efficacy of the scheme as a whole. We were also not able to assess the impact of the intervention on activities outside leisure facilities. It is also possible that the effectiveness of the scheme may also have been contingent on other factors in Blackburn with Darwen. Most notable of these is the relatively large number of leisure facility sites, many of which are in close proximity to deprived neighbourhoods. While our analysis indicates that it was likely that the intervention had an impact on physical activity, the cost effectiveness of this intervention remains uncertain.

### Implications for policy

Our study indicates that removing user charges from leisure facilities in combination with outreach and marketing activities could potentially increase overall levels of physical activity while reducing inequalities. Re:fresh may have achieved lower inequalities in participation due its universal nature, and availability of sessions during 90% of opening hours, therefore including people on low incomes who work full-time, who might be excluded from other more targeted schemes (such as the provision of cheaper facilities for those in receipt of state benefits) or only during off-peak hours.

With increasing cuts to local government budgets in the UK,^30^ many councils are considering whether to reduce the public subsidy of leisure facilities and discontinue the free leisure schemes that currently exist. There is also the potential for other funding such as local government public health grants or health service funds to be invested in subsidising leisure facilities to promote public health. Our study provides evidence that expanding free leisure schemes is likely to increase physical activity and reduce inequalities, while discontinuing these schemes may have the opposite effect.

**What is already known on this subject**

- Physical inactivity is a leading challenge for public health in the UK that costs the National Health Service an estimated £455 million per year. Rates of physical activity are lowest in more deprived populations. Interventions that improve access to public facilities, such as price reductions or free offers, have the potential to increase population-level physical activity levels, as well as addressing social inequalities in uptake of physical activity.

**What this study adds**

- This study uses quasi-experimental methods to investigate the impact of the introduction of universal free access to leisure facilities alongside community outreach activities on inequalities in physical activity. It demonstrates that this can increase participation in swimming and gym activities and overall levels of physical activity, with the effects being greatest in the most disadvantaged groups.
