# Supplementary material for: Impact of free access to leisure facilities and community outreach on inequalities in physical activity: a quasi-experimental study
Source: J Epidemiol Community Health. 2018 Jan 12;72(3):252–8. doi: 10.1136/jech-2017-209882 (PMC5868528; doi:10.1136/jech-2017-209882)
Supplement: Supplementary file 1 [file jech-2017-209882supp001.pdf]

The impact of free access to leisure facilities on inequalities in physical activity: a quasi-experimental study.

Supplementary appendices.

Appendix 1. ITS model

Interrupted time series.

Ln(gymswim)= B_{1*}re:fresh +B_2time1+B_3time2 +quarter

Where gymswim is the total number of attendances at leisure centres in Blackburn with Darwen for gym and/or swim activities in each quarter.

re:fresh is a dummy variable that is 0 before 3rd quarter of 2008 and 1 after.

Time1 is a time trend term for before the 3rd quarter of 2008, (set to zero after)

Time1 is a time trend term for after the 3rd quarter of 2008, (set to zero before)

Quarter is a set of dummy variables for the four quarters of the year.

As is shown in web Figure 3 there is some evidence of autocorrelation in the data. The regression was therefore estimated with Newey-West standard errors. We used the automatic lag selection procedure outlined by Newey and West[1] to set the maximum lag order of autocorrelation, this identified a maxim lag of 8 as appropriate. In practice as the effect size if very large this made very little difference to the findings, sensitivity analysis using other maximum lags from 1 to 8, gave results that were identical for the first two decimal places, and all p values were <0.001.

Analysis of residuals distribution and autocorrelation.

Web Figure 1 Kernal density plot of residuals – indicating that the distribution is approximately normal.

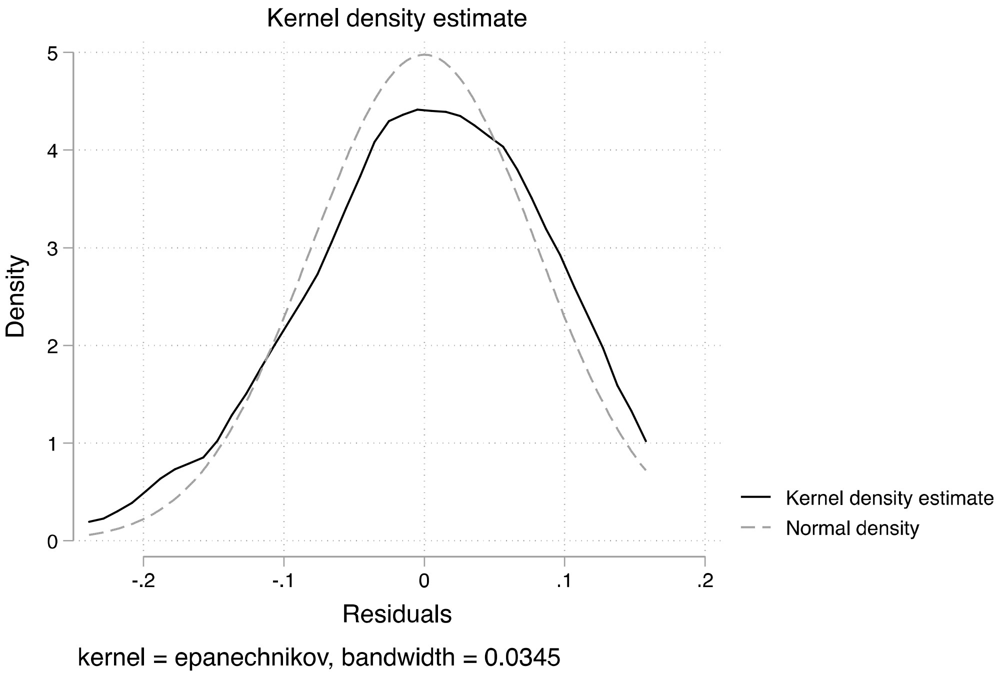

Web Figure 2 Plot of residuals over time.

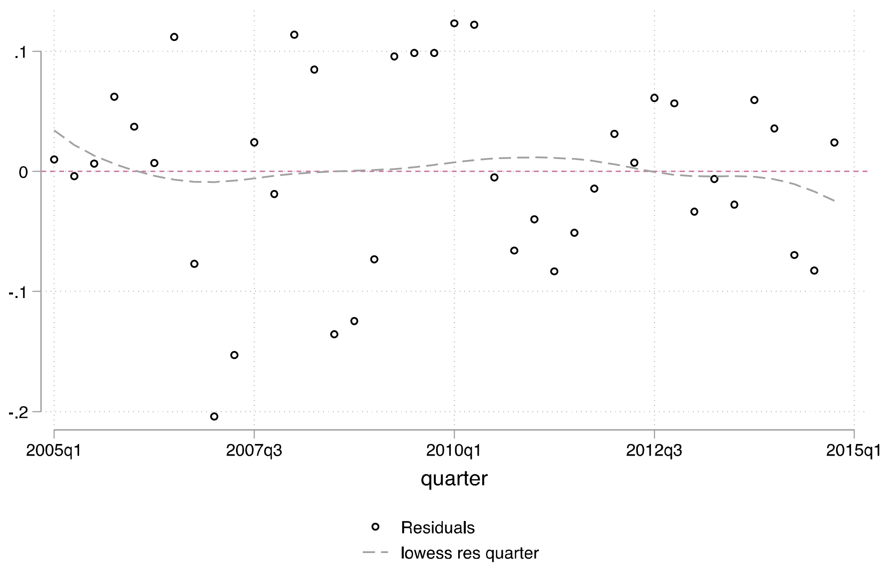

Web Figure 3 Plot showing autocorrelation of residuals.

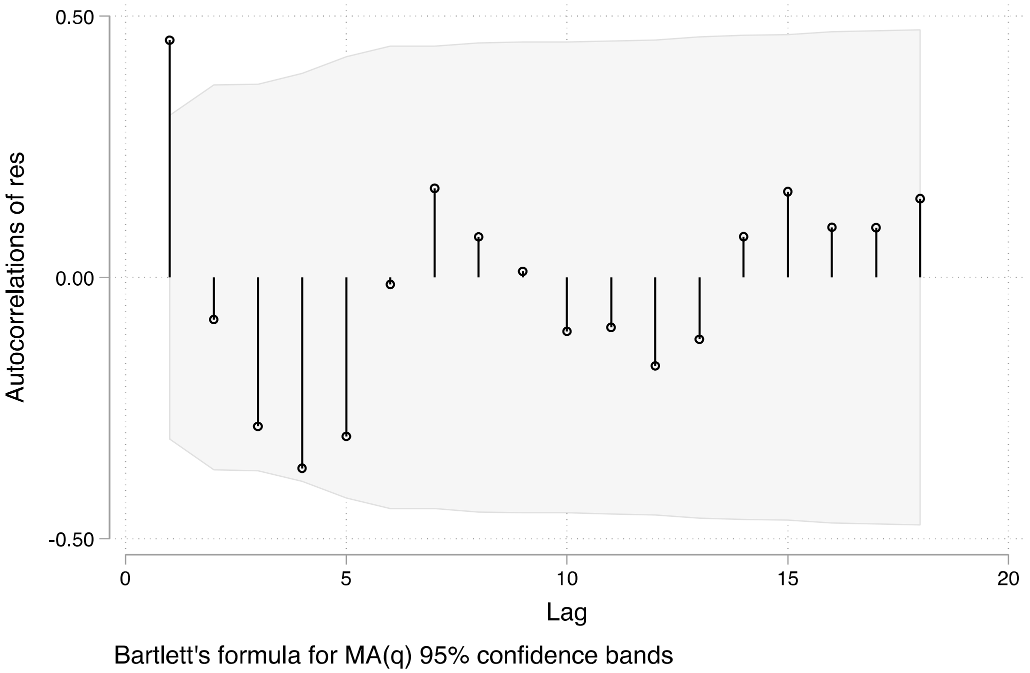

Web Figure 4 Estimated trends in gym and swimming attendances (combined) from the ITS analysis and predicted trend in the absence of the intervention.

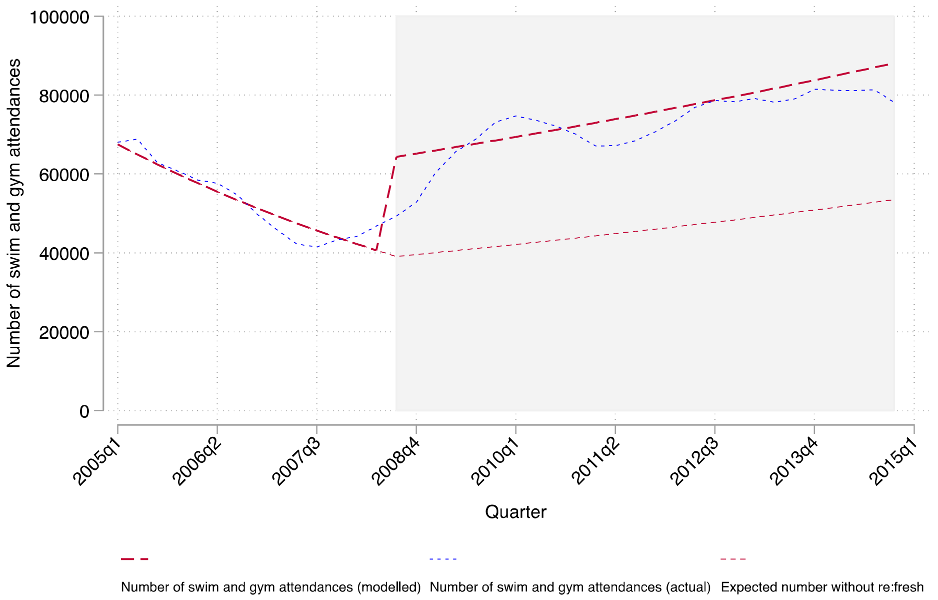

Web Figure 5 Estimated trends in gym attendances from the ITS analysis and predicted trend in the absence of the intervention.

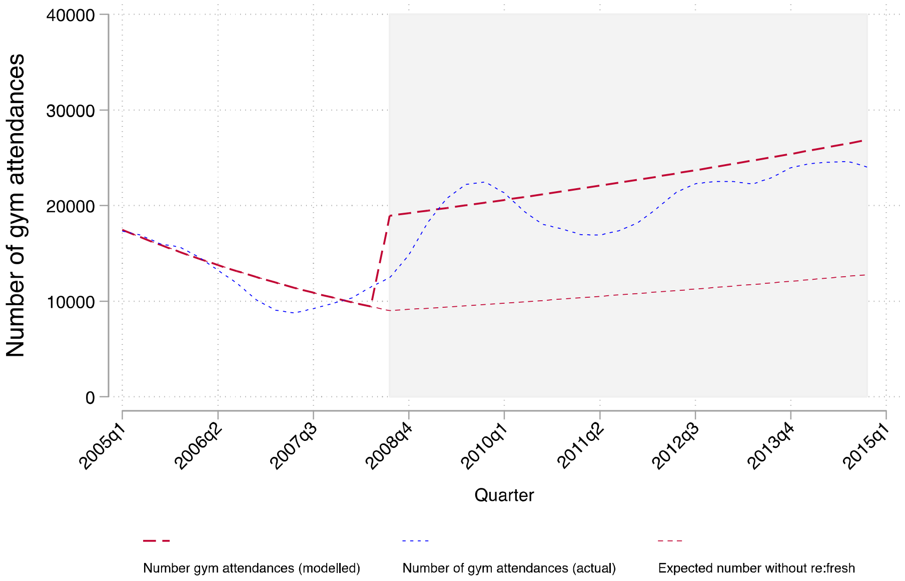

Web Figure 6 Estimated trends in swim attendances from the ITS analysis and predicted trend in the absence of the intervention.

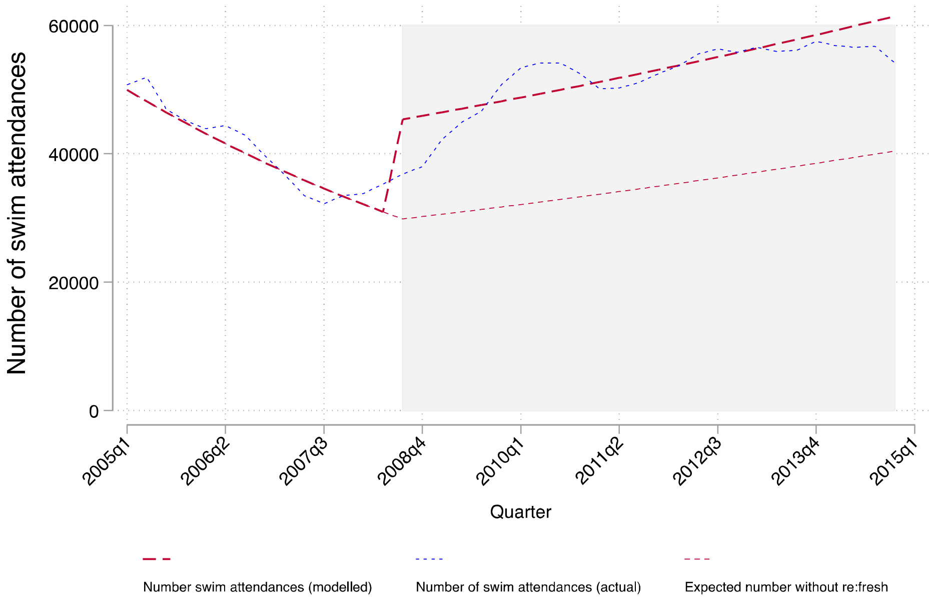

Appendix 2. Alternative model specifications.

Web Table 1 Estimated increase in swim and/or gym activity associated with the introduction of re:fresh estimated from the ITS regression analysis. % increase in activity estimated as the difference in logged number of attendances.

Outcome	Realtive increase in activity associated with introduction of re:fresh (RR)	95% CI		P -value
Gym attendances	2.1	1.76	2.5	<0.001
Swimming attendances	1.52	1.38	1.67	<0.001
Gym or swimming attendances (combined)	1.64	1.43	1.89	<0.001

Web Table 2. Absolute increase – outcome not logged transformed as in main analysis. Estimated increase in swim or gym activity associated with the introduction of re:fresh estimated from the ITS regression analysis.

Outcome	Estimated increase in quarterly activity associated with introduction of re:fresh.	95% CI		P -value
Gym or swimming attendances	26472	21011	31933	<0.001

Web Table 3.Poisson model. Estimated increase in swim or gym activity associated with the introduction of re:fresh estimated from the ITS regression analysis. Relative change in activity – relative risk.

Outcome	Estimated increase in quarterly activity associated with introduction of re:fresh (RR)	95% CI		P -value
Gym or swimming attendances	1.66	1.65	1.67	<0.001

Appendix 3. Differences in Differences. Model formula and alternative model specifications

Difference in Differences.

$Outcome_{ikt}$=$B_1 interv_k$ +$B_2 After_t$ +$B_3 After_k$*$interv_t$+$B_4 SES_{ikt}$+ $B_5 SEX_{ikt}$+ $B_6 ethnicity_{ikt}$ + $B_7 AGE_{ikt}$+ $B_8 AGESQ_{ikt}$ + B_9 $Year_t$

Where interv is a dummy variable indicating respondents in Blackburn with Darwen and is 0 otherwise.

After is a dummy variable that is 0 before 2008 and 1 after.

$After_k$*$interv_t$ is the interection between the two – B_3 if therefore the DiD parameter.

Model included survey weights to adjust for non-response. We estimated robust standard errors clustered at the local authority level to allow for within LA correlation due to sampling design.

SES is a set of dummy variables for each socioeconomic group

Ethnicity is a set of dummy variables for each ethnic group

AGE_{ikt} is the age of respondent I in local authority k at time period t.

AGESQ is the square of AGE

Year is a continuous variable indicating the survey year.

Alternative difference in differences analyses.

1. With alternative comparator groups.

Table 4. Table showing the estimates of the effect of re:fresh from the difference in difference analysis – comparing results using all other LAs in England as the comparison group (as reported on the paper), as compared to restricting the comparison group to similarly deprived LAs.

Outcome	Comparator group	Percentage point increase in outcome associated with introduction of re:fresh	95% CI	
% Participating in at least 30 minutes of moderate intensity gym or swim sessions in a month	325 other LAs in England	3.8	3.7	4
	42 other deprived LAs (bottom quintile)	3.0	2.6	3.4
	30 Other deprived LAs outside London	2.8	2.4	3.3
	13 other deprived LAs outside London with high BME population (highest quintile)	2.9	2.2	3.7
	15 other deprived LAs in the North West	2.8	2.0	3.7
% participating any sport or active recreation of at least moderate intensity for at least 30 minutes on at least 12 days out of the last 4 weeks	325 other LAs in England	1.8	1.6	2
	42 other deprived LAs (bottom quintile)	1.8	1.2	2.3
	30 Other deprived LAs outside London	1.4	0.8	2.0

	13 other deprived LAs outside London with high BME population (highest quintile)	1.4	0.2	2.5
	15 other deprived LAs in the North West	1.5	0.5	2.4

2. With intervention period set to start from 2009 rather than 2008

Table 5. Table showing the estimates of the effect of re:fresh from the difference in difference analysis – With intervention period set to start from 2009 rather than 2008.

Outcome	Percentage point increase in outcome associated with introduction of re:fresh	95% CI	
% Participating in at least 30 minutes of moderate intensity gym or swim sessions in a month	3.9	3.7	4.1
% participating any sport or active recreation of at least moderate intensity for at least 30 minutes on at least 12 days out of the last 4 weeks	0.9	0.8	1.1

3. Separate analysis for gym and swimming participation.

Web Figure 7 . Estimates of the effect of the introduction of re:fresh from the difference-in-differences analysis on (1) % participating in gym activity and (2) % participating in swim activity at least once in the past month Results for all socioeconomic groups in Blackburn with Darwen and separately for 3 socioeconomic groups. Effect sizes indicate the additional percentage of the population participating due to the intervention.

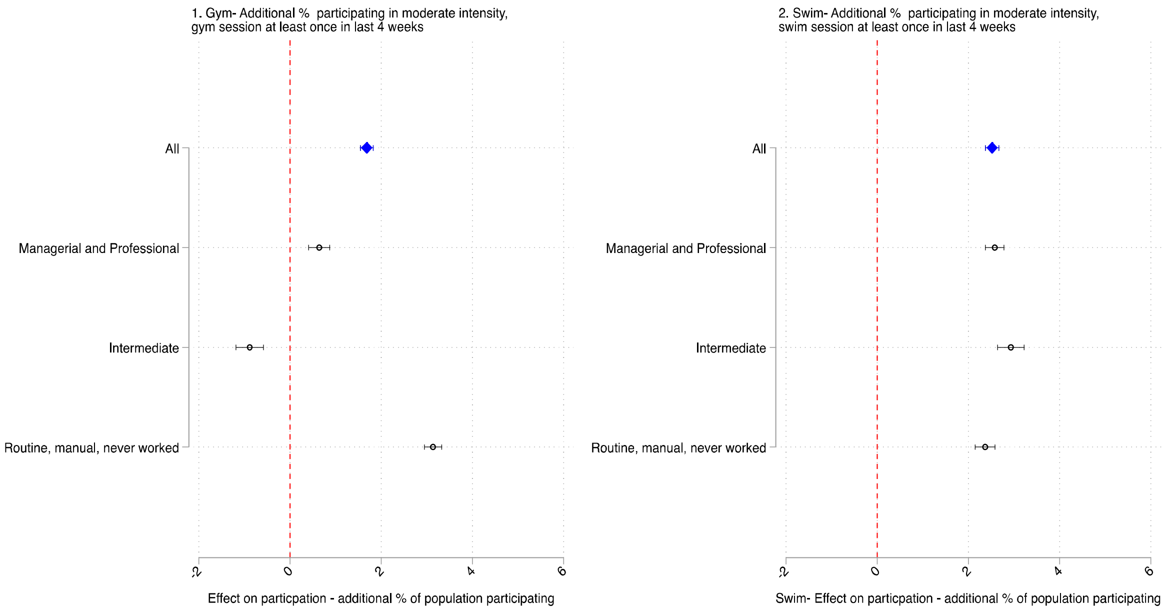

4. Analysis by socioeconomic group with never worked removed from lowest socioeconomic group.

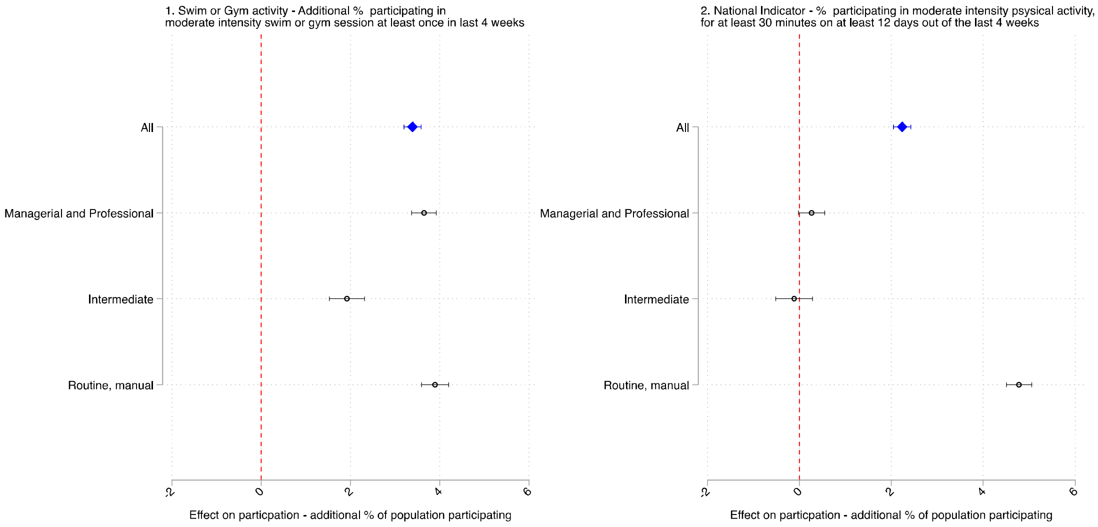

Appendix 4. Inequalities in participation before and after intervention by socioeconomic group.

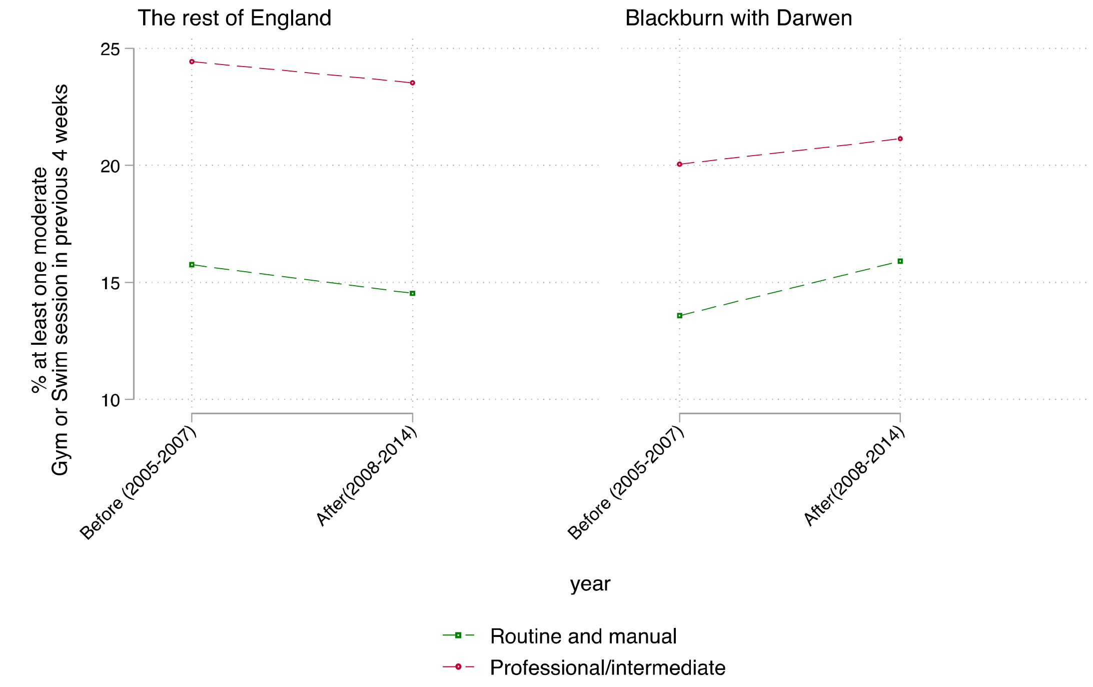

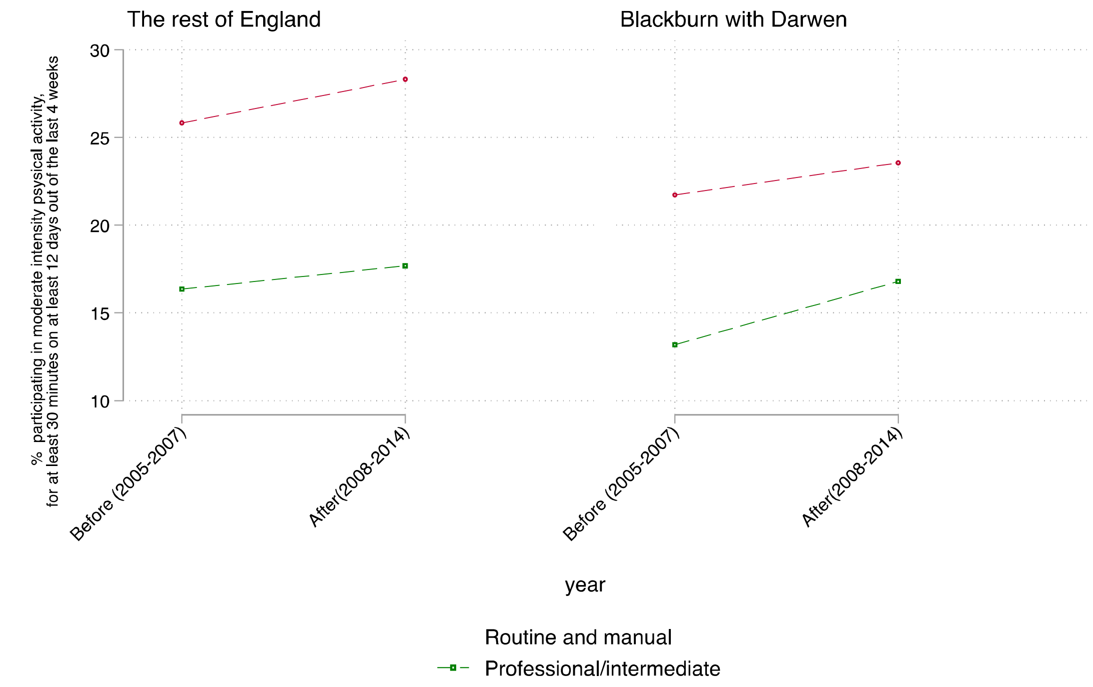

Appendix 5. Map of Blackburn with Darwen showing distribution of facilities in relation to area deprivation.

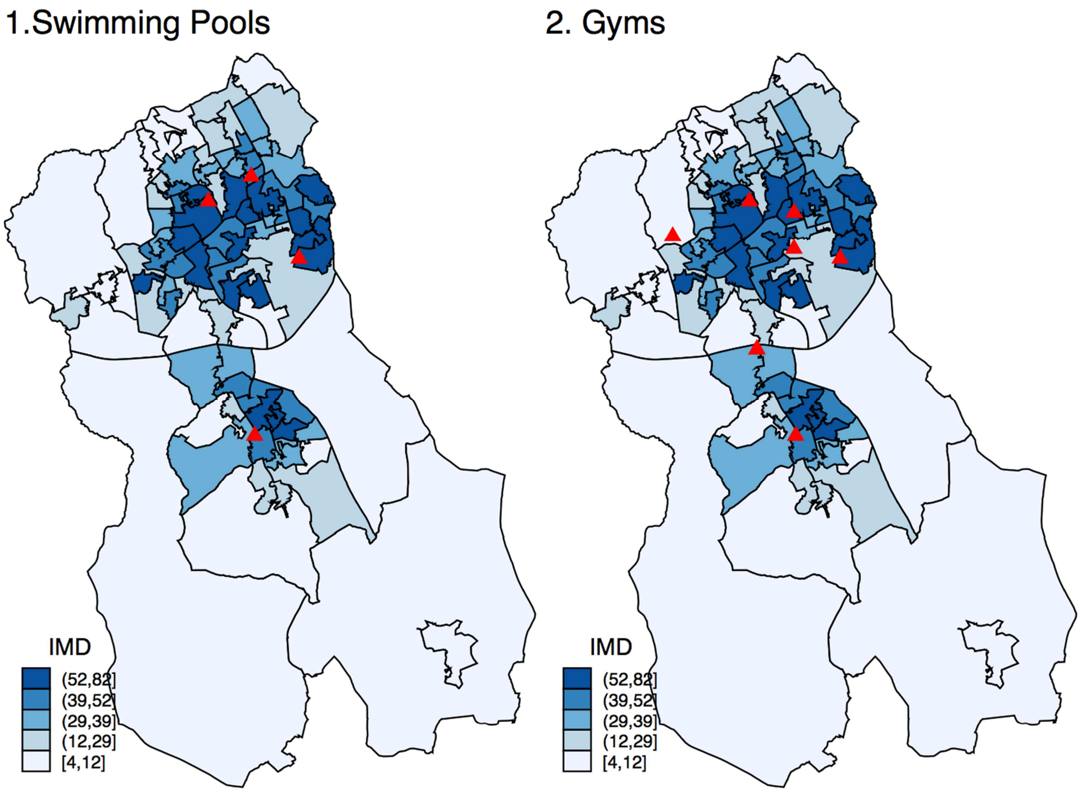

Appendix 6. Comparison between Swimming and Gym rates in the Active Peoples Survey and the Health Survey for England.

Table 6 shows the rates of swimming and gym related activity in the Active Peoples Survey and the Health Survey for England in 2008 showing very similar rates between the two surveys.

Web Table 6.

	**Any swimming in the past 4 weeks (95% CI)**	**Any gym/Exercise bike/Weight training activity in the past 4 weeks**
Active People Survey (2008-09)	13.3%(13.0 to 13.5)	14.0% (13.8 to 14.2)
Health Survey for England 2008.	13.6% (13.0 to 14.1)	14.6% (14.0 to 15.3)

Active Peoples Survey

Q9. So thinking about *the last four weeks, that is since [^INSERT^]*, did you do any sporting or recreational physical activity?

1. Yes
2. No
3. Don't know

Asked if Q9==1

Q10. What have you done? DO NOT PROMPT. CODE ALL MENTIONED. WHERE A DATABASE SEARCH BRINGS UP A NUMBER OF ACTIVITIES FOR A SPORT PLEASE PROBE CAREFULLY FOR THE EXACT ACTIVITY UNDERTAKEN. IF ACTIVITY NOT ON DATABASE CODE OTHER AND ENTER AS OTHER SPECIFY.

Health Survey for England.

ActPhy: Can you tell me if you have done any activities on this card during the last 4 weeks, that is since *(date of interview – 4 weeks)*? Please include teaching, coaching, training and practice sessions.

1 Yes 2 No

IF ActPhy = Yes THEN WhtAct

WhtAct : Which have you done in the last four weeks? PROBE: Any others? CODE ALL THAT APPLY.

1 Swimming

2 Cycling

3 Workout at a gym/Exercise bike/Weight training

4 Aerobics/Keep fit/Gymnastics/ Dance for fitness

5 Any other type of dancing

6 Running/Jogging

7 Football/Rugby

8 Badminton/tennis

9 Squash

10 Exercises (e.g. press-up, sit-ups).
